# Overexpression of thioredoxin-like protein ACHT2 leads to negative feedback control of photosynthesis in *Arabidopsis thaliana*

**DOI:** 10.1007/s10265-024-01519-2

**Published:** 2024-02-17

**Authors:** Yuka Fukushi, Yuichi Yokochi, Toru Hisabori, Keisuke Yoshida

**Affiliations:** 1 Laboratory for Chemistry and Life Science, Institute of Innovative Research, Tokyo Institute of Technology, Yokohama, 226-8501 Japan; 2 School of Life Science and Technology, Tokyo Institute of Technology, Yokohama, 226-8501 Japan; 3 International Research Frontier Initiative, Tokyo Institute of Technology, Yokohama, 226-8501 Japan

**Keywords:** Chloroplast, Non-photochemical quenching (NPQ) regulation, Redox regulation, Thioredoxin, Thioredoxin-like protein

## Abstract

Thioredoxin (Trx) is a small redox mediator protein involved in the regulation of various chloroplast functions by modulating the redox state of Trx target proteins in ever-changing light environments. Using reducing equivalents produced by the photosynthetic electron transport chain, Trx reduces the disulfide bonds on target proteins and generally turns on their activities. While the details of the protein-reduction mechanism by Trx have been well investigated, the oxidation mechanism that counteracts it has long been unclear. We have recently demonstrated that Trx-like proteins such as Trx-like2 and atypical Cys His-rich Trx (ACHT) can function as protein oxidation factors in chloroplasts. Our latest study on transgenic Arabidopsis plants indicated that the ACHT isoform ACHT2 is involved in regulating the thermal dissipation of light energy. To understand the role of ACHT2 in vivo, we characterized phenotypic changes specifically caused by ACHT2 overexpression in Arabidopsis. ACHT2-overexpressing plants showed growth defects, especially under high light conditions. This growth phenotype was accompanied with the impaired reductive activation of Calvin–Benson cycle enzymes, enhanced thermal dissipation of light energy, and decreased photosystem II activity. Overall, ACHT2 overexpression promoted protein oxidation that led to the inadequate activation of Calvin–Benson cycle enzymes in light and consequently induced negative feedback control of the photosynthetic electron transport chain. This study highlights the importance of the balance between protein reduction and oxidation in chloroplasts for optimal photosynthetic performance and plant growth.

## Introduction

Plants have various mechanisms for surviving in ever-changing environments. One such mechanism is redox regulation in chloroplasts, which reversibly modulates the reduction and oxidation states of target proteins and thus their enzymatic activities. The key protein involved in redox regulation is thioredoxin (Trx). In plant chloroplasts, Trx reduces its target proteins through the reducing power generated by the light-driven photosynthetic electron transport chain and transmitted from ferredoxin via ferredoxin/thioredoxin reductase (Buchanan 1980; Buchanan et al. 2002). Trx transfers reducing power to target proteins through a dithiol–disulfide exchange reaction using a pair of cysteine residues in the active site of WCGPC. Most Trx target proteins are inactivated in their oxidized form and activated in their reduced form. Trx target proteins are involved in several chloroplast functions, including the Calvin–Benson cycle, ATP synthesis, antioxidant system, and chloroplast biogenesis (Yoshida and Hisabori 2023). Therefore, the redox regulation system may regulate various functions in chloroplasts in response to changes in light environment by linking photosynthetic electron transfer reactions and metabolic reactions.

Trx in chloroplasts is classified into five subtypes, Trx-*f*, -*m*, -*x*, -*y*, and -*z* (Lemaire et al. 2007; Serrato et al. 2013). The subtypes differ in molecular properties, such as redox potential, protein surface charge, and target-recognition residues, which determine their target protein selectivity (Collin et al. 2003; Le Moigne et al. 2021; Toivola et al. 2013; Yokochi et al. 2019; Yoshida et al. 2015). For example, Calvin–Benson cycle enzymes are mainly reduced by Trx-*f* and Trx-*m* (Michelet et al. 2013; Yoshida et al. 2015). Trx-*m* may also be involved in the control of cyclic electron transport around photosystem (PS) I (Okegawa and Motohashi 2020). Our findings in a recent study also indicate that redox regulation is physiologically essential for plants. Arabidopsis expressing the chloroplast NADP-malate dehydrogenase variant, which was modified into a stable active form by deleting the redox-regulated cysteine, showed growth inhibition under fluctuating light conditions (Yokochi et al. 2021a). Thus, redox regulation is significant for its ability to appropriately oxidize the enzyme and switch its function off under dark or limited light conditions, which supports optimal plant growth. Despite this important function, the mechanisms of the oxidation side of the redox regulation system have remained unclear.

Recently, we found that Trx-like2 (TrxL2) and atypical Cys His-rich Trx (ACHT) are responsible for oxidizing Trx target proteins (Yokochi et al. 2021b, 2019; Yoshida et al. 2018, 2019a, b). TrxL2 and ACHT are classified as Trx-like proteins and have active site sequences similar to Trx, which are WCRKC and WCG/ASC, respectively. They are characterized by a higher redox potential and a higher efficiency in reducing 2-Cys peroxiredoxins (2-Cys Prx) than typical Trx (Dangoor et al. 2009, 2012; Yokochi et al. 2019; Yoshida et al. 2018). The 2-Cys Prx uses reducing equivalents to reductively detoxify hydrogen peroxide (H_2_O_2_). Therefore, TrxL2 and ACHT are expected to continuously oxidize proteins under light conditions where H_2_O_2_ is produced as a byproduct of photosynthesis (Asada 2006).

In Arabidopsis, TrxL2 and ACHT have two (TrxL2.1 and TrxL2.2) and five (ACHT1 to ACHT5) isoforms, respectively, each of which shows different tissue-specific expression patterns (Belin et al. 2015; Chibani et al. 2009). They also have different target oxidation selectivities; TrxL2 mainly oxidizes chloroplast ATP synthase γ-subunit (CF_1_-γ), whereas ACHT mainly oxidizes fructose-1,6-bisphosphatase (FBPase) (Sekiguchi et al. 2022; Yokochi et al. 2021b). However, functional differences among Trx-like proteins have not been elucidated. Notably, ACHT2-overexpressing plants showed growth defects and high non-photochemical quenching (NPQ), the mechanism that dissipates excess light energy as heat (Yokochi et al. 2021b). This finding raises the possibility of negative impacts of ACHT2 on plants.

In this study, we therefore aimed to clarify the physiological role of ACHT2 by characterizing the detailed phenotypes of ACHT2-overexpressing plants. Our data provide important insights into the physiological consequences of the imbalance in the protein redox states during photosynthesis.

## Materials and methods

### Plant materials and growth conditions

*Arabidopsis thaliana* (Col-0) was used as the control and was designated as WT. ACHT2-overexpressing plant lines generated in the previous study and designated as ACHT2-TF1 and ACHT2-TF4 (Yokochi et al. 2021b) were renamed ACHT2-OE (ACHT2-OE1 and ACHT2-OE2, respectively) in the present study. WT and ACHT2-OE plants were grown in soil under a 16-h light (20, 60, or 650 µmol photons m^−2^ s^−1^)/8-h dark cycle at 22 °C for 4 weeks (20 or 60 µmol photons m^−2^ s^−1^) or 3 weeks (650 µmol photons m^−2^ s^−1^).

### Measurement of fresh weight and chlorophyll content

Fresh weight was measured using the aboveground portion of the plants. The chlorophyll content in rosette leaves was determined as the sum of the contents of chlorophyll a and b after extraction with 80% (v/v) acetone, as described in a previously published method (Porra et al. 1989).

### Determination of the light-dependent protein redox state in vivo

Plants grown at a light intensity of 60 µmol photons m^−2^ s^−1^ were dark-adapted for 8 h and then irradiated. The plant leaves were harvested at the indicated times and frozen in liquid nitrogen. The redox states of the proteins in plant leaves were determined as described previously (Yoshida et al. 2014). The anti-FBPase and anti-CF_1_-γ antibodies were prepared as described previously (Konno et al. 2012; Yoshida et al. 2014). The anti-RCA antibody was commercially procured (catalog no. AS10-700, Agrisera, Vännäs, Sweden).

### Measurement of photosynthetic parameters

Plants grown at a light intensity of 60 µmol photons m^−2^ s^−1^ were dark-adapted for 8 h, after which Fv/Fm, Y(II), and NPQ were measured using a Dual-PAM-100 spectrometer (Walz, Heinz, Germany). The time courses of Y(II) and NPQ were measured with actinic red light at 60 µmol photons m^−2^ s^−1^ for 8 min, while recovery in darkness was recorded for 8 min. Saturating pulses of red light were applied at 6000 µmol photons m^−2^ s^−1^ at 0.4-s durations. Y(II) and NPQ were calculated on the DUAL-PAM-100 software using previously applied equations (Kramer et al. 2004).

### Extraction and quantitative analysis of xanthophyll cycle pigments

Plants grown at a light intensity of 60 µmol photons m^−2^ s^−1^ were dark-adapted for 8 h, irradiated at 60 µmol photons m^−2^ s^−1^ for 30 min, and retuned to dark conditions. Leaves were detached at the indicated times and frozen in liquid nitrogen. Pigments were extracted by grinding 20–30 mg leaves in liquid nitrogen, and the resulting leaf powder was suspended with 600 µL 100% acetone. Quantitative analysis of the xanthophyll cycle pigments (violaxanthin, antheraxanthin and zeaxanthin) was performed using HPLC as previously described (Muller-Moule et al. 2002).

## Results and discussion

### Overexpression of ACHT2 induced growth defects

**Fig. 1:**
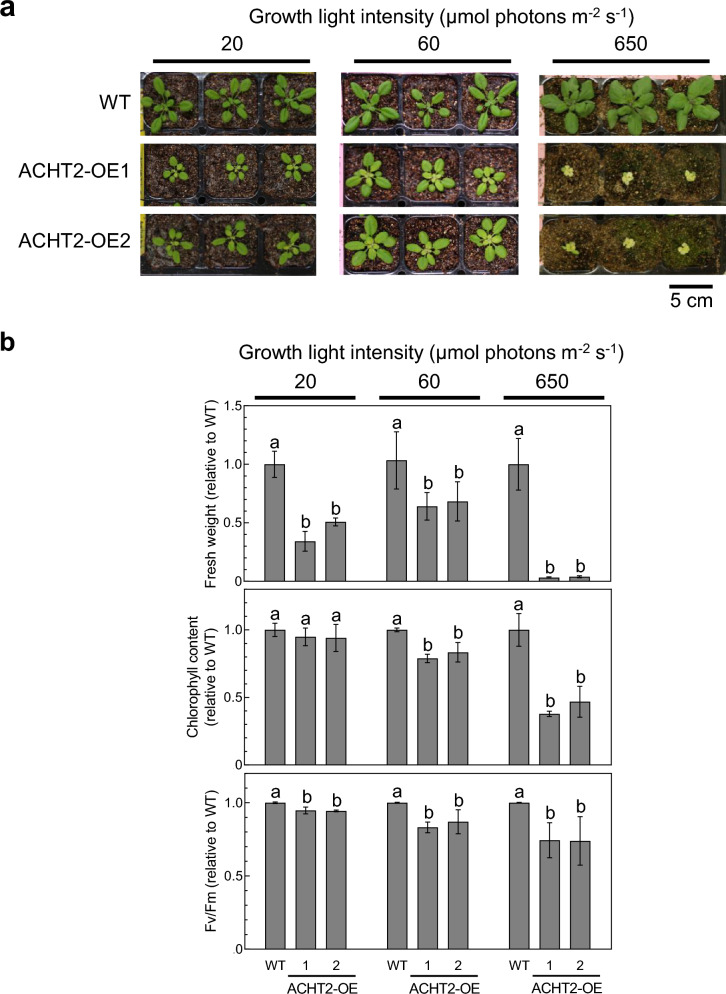
Growth phenotypes of ACHT2-OE plants. **a** ACHT2-OE plants grown at different growth light intensities; 20, 60, or 650 µmol photons m^−2^ s^−1^. Plants were grown under long day conditions (16-h light/8-h dark) for 4 weeks (20 or 60 µmol photons m^−2^ s^−1^) or 3 weeks (650 µmol photons m^−2^ s.^−1^). **b** Fresh weight, chlorophyll content, and Fv/Fm. The data are shown relative to WT values. Each value represents the mean ± SD (*n* = 3 to 11). Different letters indicate significant differences among plants (*P* < 0.05; one-way ANOVA and Tukey’s HSD)

In our previous study, we obtained four transgenic Arabidopsis plants (ACHT2-TF1 to ACHT2-TF4; “TF” denoting “transformed”) with various levels of ACHT2 overexpression (Yokochi et al. 2021b). Of these, ACHT2-TF1 and ACHT2-TF4 showed high expression of ACHT2 (ACHT2-TF1, 25-fold of WT; ACHT-TF4, 16-fold of WT). In the present study, we renamed ACHT2-TF to ACHT2-OE; ACHT2-TF1 to ACHT2-OE1; and ACHT2-TF4 to ACHT2-OE2. We then analyzed the fresh weight, chlorophyll content, and maximal quantum yield of PSII (Fv/Fm) in ACHT2-OE plants under various light intensities (20, 60, and 650 µmol photons m^−2^ s^−1^) (Fig. 1). The fresh weights and Fv/Fm of ACHT2-OE plants were lower than those of WT under all light conditions. Notably, the phenotypic changes in ACHT2-OE plants were more significant under higher light conditions; for example, the fresh weight of ACHT2-OE plants were less than 5% of that of WT. These results show that the effects of ACHT2 overexpression on the growth phenotype may be correlated with light intensity. Plants grown at a light intensity of 60 µmol photons m^−2^ s^−1^ were used for subsequent experiments.

### Overexpression of ACHT2 altered reduction levels of Trx target proteins

Next, we examined the redox status of certain Trx target proteins in ACHT2-OE plants under light conditions. Changes in the redox states of Trx target proteins CF_1_-γ, FBPase, and Rubisco activase (RCA) were determined by thiol modification using 4-acetamido-4’-maleimidyl-stilbene-2,2’-disulfonate.

**Fig. 2:**
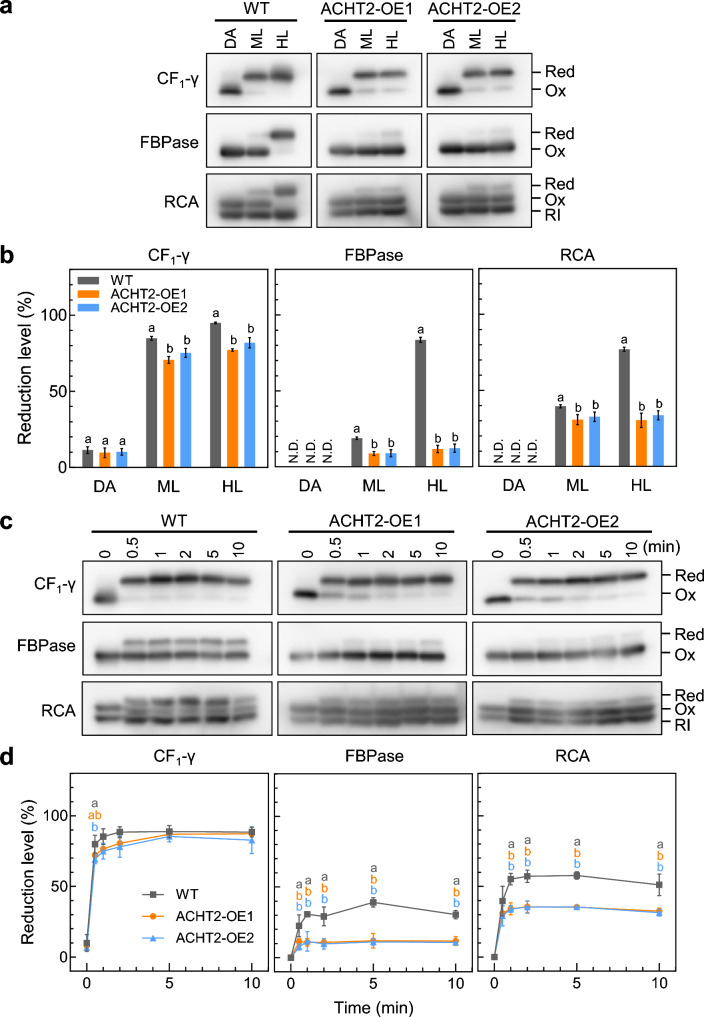
In vivo redox responses of Trx target proteins in ACHT2-OE plants. Dark-adapted plants were placed under the indicated light conditions. **a** Western blotting image of the detection of the redox state of CF_1_-γ, FBPase, and RCA after 8 h of dark adaptation (DA) or 30 min of light irradiation at 60 µmol photons m^−2^ s^−1^ (ML) or 650 µmol photons m^−2^ s^−1^ (HL). **b** Reduction levels of CF_1_-γ, FBPase, and RCA based on the signal intensities shown in (a). **c** Western blotting image of the detection of the redox state of CF_1_-γ, FBPase, and RCA at 0–10 min of light irradiation at 60 µmol photons m^−2^ s.^−1^. **d** Reduction levels of CF_1_-γ, FBPase, and RCA based on the signal intensities shown in (**c**). **b, d** The reduction level was determined as the ratio of the reduced form to the total amount of reduced and oxidized forms. Each value represents the mean ± SD (*n* = 3). *Red* reduced form, *Ox* oxidized form, *RI* redox-insensitive splicing variant. Different letters indicate significant differences among plants (*P* < 0.05; one-way ANOVA and Tukey’s HSD)

Figure 2a and b show the reduction levels of Trx target proteins at steady-state photosynthesis, measured after 30 min irradiation of moderate or high light (60 or 650 µmol photons m^−2^ s^−1^). In ACHT2-OE plants, the reduction levels of CF_1_-γ, FBPase, and RCA were lower than those in WT under both light conditions. Especially, the reduction levels of FBPase and RCA were largely lowered under high light conditions (about 15% and 40% of that of WT, respectively). Figure 2c and d show the reduction patterns of Trx target proteins during the dark-to-light (60 µmol photons m^−2^ s^−1^) transitions. In WT, CF_1_-γ, FBPase, and RCA were reduced to saturated reduction levels after 1–2 min light irradiation. In ACHT2-OE plants, the reduction levels of FBPase and RCA were saturated at lower levels. Their reduction kinetics after irradiation were apparently comparable with those of WT, although we need more detailed analyses to discuss the reduction kinetics. Taken together, ACHT2 overexpression lowers the steady-state reduction level of Trx target proteins under light conditions.

We previously found that the knockout of ACHT2 leads to the delayed oxidation of FBPase during the light-to-dark transitions (Yokochi et al. 2021b). This result is in agreement with the present finding of the impaired reduction of FBPase in ACHT2-OE plants (Fig. 2), which strongly suggests that ACHT2 acts as the major oxidation factor for FBPase in vivo. By contrast, the contribution of ACHT2 to RCA oxidation is still unclear. ACHT2 knockout did not affect the oxidation process of RCA significantly (Yokochi et al. 2021b), while ACHT2 overexpression caused the impaired reduction of RCA (Fig. 2). These results indicate that ACHT2 has an ability to oxidize RCA, but its role can be complemented by other Trx and Trx-like proteins. In line with this idea, our previous study suggested that Trx-*f* is the most dominant factor for RCA oxidation (Yokochi et al. 2021b).

The reduced forms of FBPase and RCA are enzymatically active (Buchanan 1980; Michelet et al 2013). It is thus possible that lower reduction levels of FBPase and RCA in ACHT2-OE plants (Fig. 2) result in suppression of the Calvin–Benson cycle. Some growth parameters, including the fresh weight and chlorophyll content, were also negatively affected in ACHT-OE2 plants (Fig. 1). These phenotypic changes were more pronounced under high light conditions. Therefore, the inadequate functioning of the Calvin–Benson cycle caused by ACHT2 overexpression may at least partly account for the growth impairment in ACHT2-OE plants.

### Overexpression of ACHT2 induced high NPQ

**Fig. 3:**
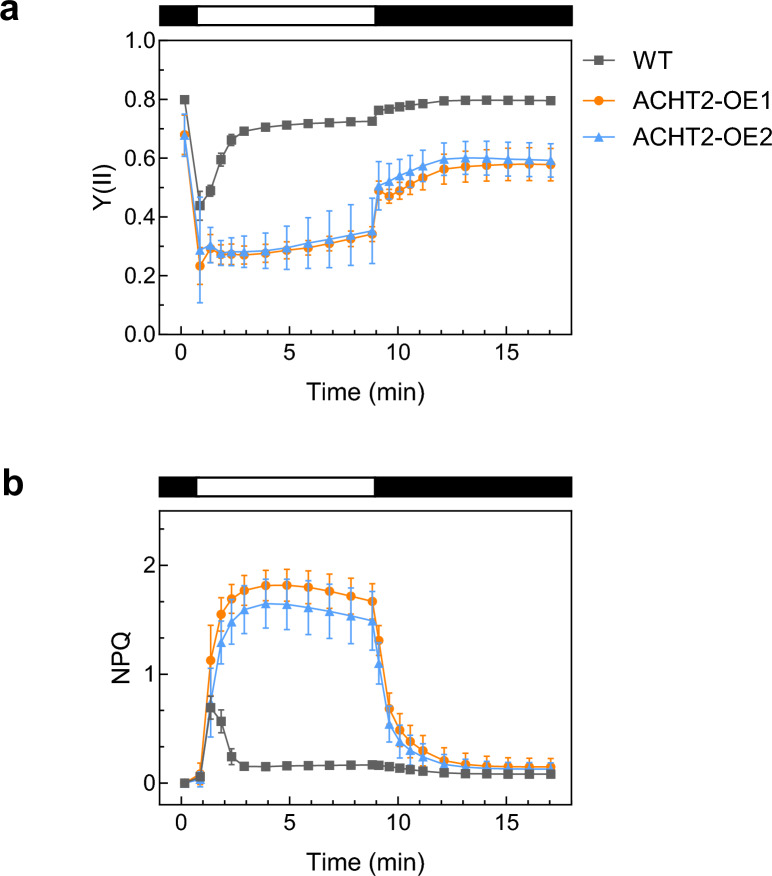
Chlorophyll fluorescence parameters in ACHT2-OE plants. Chlorophyll fluorescence was measured using a pulse-amplitude modulation fluorometer. After 8 h of dark adaptation, chlorophyll fluorescence parameters were measured under illumination at 60 µmol photons m^−2^ s.^−1^ for 8 min, followed by 8 min of darkness. White and black bars above each graph indicate periods of illumination and darkness, respectively. Time courses of Y(II) **(a)** and NPQ **(b)**. Each value represents the mean ± SD (*n* = 4–5)

To further assess the effects of ACHT2 overexpression, we measured the effective quantum yield of PSII [Y(II)] and NPQ in ACHT2-OE plants (Fig. 3). NPQ reflects the extent of the thermal dissipation of excess energy around PSII. In WT, Y(II) immediately decreased after light irradiation; however, it then increased and reached a steady state within 3–4 min. In contrast, ACHT2-OE plants showed lower Y(II) levels during light irradiation than WT (Fig. 3a). The NPQ value in WT is transiently increased after 1 min of light irradiation but then decreased to a low level. In contrast, ACHT2-OE plants maintained a much higher NPQ than WT (Fig. 3b). Thus, ACHT2 overexpression lowered steady-state Y(II) and enhanced NPQ induction.

**Fig. 4:**
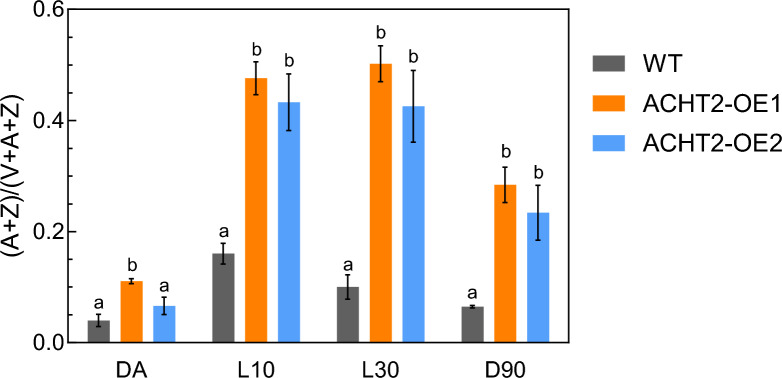
Composition of xanthophyll cycle pigments in ACHT2-OE plants. After 8 h of dark adaptation, plants were placed under light conditions (60 µmol photons m^−2^ s.^−1^) and then placed in darkness. The violaxanthin (V), antheraxanthin (A), and zeaxanthin (Z) contents were determined by HPLC after 8 h of dark adaptation (DA), after 10 min (L10) or 30 min (L30) light irradiation, and after a subsequent return to darkness for 90 min (D90). Data are expressed as the ratio of the sum of antheraxanthin and zeaxanthin to the total amount of xanthophyll cycle pigments. Each value represents the mean ± SD (*n* = 3). Different letters indicate significant differences among plants (*P* < 0.05; one-way ANOVA and Tukey’s HSD)

NPQ consists of several components, the main one being qE, which is characterized by fast induction and relaxation kinetics within a few minutes after light irradiation and returning to dark conditions, respectively (Nilkens et al. 2010; Ruban 2016). qE is induced by the protonation of PSII subunit PsbS and the conversion of pigments in the xanthophyll cycle (Li et al. 2000; Niyogi et al. 1998). In the xanthophyll cycle, violaxanthin is converted to zeaxanthin via antheraxanthin by violaxanthin de-epoxidase, which is activated by lumen acidification (Szabo et al. 2005). Under dark conditions, zeaxanthin is reconverted to violaxanthin by zeaxanthin epoxidase. To uncover why ACHT2-overexpressing plants exhibited high NPQ, we determined the composition of the xanthophyll cycle pigments (Fig. 4). Plants were collected for the HPLC analysis after 8 h of dark, after 10 and 30 min of light irradiation at 60 µmol photons m^−2^ s^−1^, and after 90 min of returning to dark conditions. ACHT2-OE plants showed a 3- to 4-fold higher ratio of antheraxanthin and zeaxanthin to total xanthophyll pigment contents during light irradiation than WT. Thus, the high NPQ phenotype of ACHT2-OE plants must be attributed to the high de-epoxidation state of the xanthophyll cycle.

### Possible consequences of ACHT2 overexpression in vivo

We investigated the physiological impacts of ACHT2 overexpression, which is suggested to be a protein oxidation factor in chloroplasts. ACHT2 overexpression resulted in impaired plant growth, impaired protein reduction, lowered PSII activity, and elevated NPQ (Figs. 1, 2, 3).

When the activity of the Calvin-Benson cycle is low, the supply of energy (ATP or NADPH) exceeds its requirement in photosynthesis. The photoinhibition of PSII will likely be accelerated under such conditions (Takahashi and Murata 2005). In this case, NPQ (qE) is activated by lumen acidification induced by cyclic electron transport around PSI to protect PSII from excess light energy (Ruban et al. 2012; Szabo et al. 2005). For instance, the chemical inhibition of Calvin–Benson cycle enzymes results in high NPQ induction and slow linear electron transport (Joliot and Alric 2013). The high NPQ in ACHT2-OE plants was accompanied by a high de-epoxidation state of the xanthophyll cycle (Fig. 4), indicating increased qE levels. Hence, the lower Y(II) and higher NPQ observed in ACHT2-OE plants are caused by negative feedback regulation resulting from the decreased function of Calvin–Benson cycle enzymes due to ACHT2 overexpression.

What is the main cause of the growth defect in ACHT2-OE plants? It may be the suppression of Calvin-Benson cycle activity and the resulting decrease in photosynthetic carbon fixation. The induction of excessive NPQ (Fig. 3) and the photoinhibition of PSII (Fig. 1b) can be considered as other possible causes. Furthermore, it is also conceivable that these factors caused the growth defect in a combined manner. Further studies are needed to clarify the mechanisms underlying the growth defect in ACHT2-OE plants. Notably, Naranjo et al. (2016) used the PsbS-deficient *npq4* mutant to test the involvement of excessive NPQ induction in the growth defect observed in the *ntrc* mutant. Accordingly, it is worth trying to cross the *npq4* mutant with ACHT2-OE plants and characterize the growth and NPQ phenotypes of the resulting plants.

In conclusion, this study showed that the redox imbalance in Trx target proteins in chloroplasts was caused by the enhancement of protein thiol oxidation, which decreased photosynthetic activity, ultimately leading to growth defects in plants. The protein-oxidizing pathway always functions during photosynthesis; thus, under such conditions, the redox state of Trx target proteins should be suitably balanced by the cooperative interaction between protein reduction and oxidation pathways for optimal plant growth.

## Data Availability

The data that support the findings of this study are available from the corresponding author upon reasonable request.

## References

[CR1] Asada K (2006). Production and scavenging of reactive oxygen species in chloroplasts and their functions. Plant Physiol.

[CR2] Belin C, Bashandy T, Cela J, Delorme-Hinoux V, Riondet C, Reichheld JP (2015). A comprehensive study of thiol reduction gene expression under stress conditions in *Arabidopsis thaliana*. Plant Cell Environ.

[CR3] Buchanan BB (1980). Role of light in the regulation of chloroplast enzymes. Annu Rev Plant Physiol.

[CR4] Buchanan BB, Schurmann P, Wolosiuk RA, Jacquot JP (2002). The ferredoxin/thioredoxin system: from discovery to molecular structures and beyond. Photosynth Res.

[CR5] Chibani K, Wingsle G, Jacquot JP, Gelhaye E, Rouhier N (2009). Comparative genomic study of the thioredoxin family in photosynthetic organisms with emphasis on *Populus trichocarpa*. Mol Plant.

[CR6] Collin V, Issakidis-Bourguet E, Marchand C, Hirasawa M, Lancelin JM, Knaff DB, Miginiac-Maslow M (2003). The Arabidopsis plastidial thioredoxins: new functions and new insights into specificity. J Biol Chem.

[CR7] Dangoor I, Peled-Zehavi H, Levitan A, Pasand O, Danon A (2009). A small family of chloroplast atypical thioredoxins. Plant Physiol.

[CR8] Dangoor I, Peled-Zehavi H, Wittenberg G, Danon A (2012). A chloroplast light-regulated oxidative sensor for moderate light intensity in Arabidopsis. Plant Cell.

[CR9] Joliot P, Alric J (2013). Inhibition of CO_2_ fixation by iodoacetamide stimulates cyclic electron flow and non-photochemical quenching upon far-red illumination. Photosynth Res.

[CR10] Konno H, Nakane T, Yoshida M, Ueoka-Nakanishi H, Hara S, Hisabori T (2012). Thiol modulation of the chloroplast ATP synthase is dependent on the energization of thylakoid membranes. Plant Cell Physiol.

[CR11] Kramer DM, Johnson G, Kiirats O, Edwards GE (2004). New fluorescence parameters for the determination of Q_A_ redox state and excitation energy fluxes. Photosynth Res.

[CR12] Le Moigne T, Gurrieri L, Crozet P, Marchand CH, Zaffagnini M, Sparla F, Lemaire SD, Henri J (2021). Crystal structure of chloroplastic thioredoxin z defines a type-specific target recognition. Plant J.

[CR13] Lemaire SD, Michelet L, Zaffagnini M, Massot V, Issakidis-Bourguet E (2007). Thioredoxins in chloroplasts. Curr Genet.

[CR14] Li XP, Bjorkman O, Shih C, Grossman AR, Rosenquist M, Jansson S, Niyogi KK (2000). A pigment-binding protein essential for regulation of photosynthetic light harvesting. Nature.

[CR15] Michelet L, Zaffagnini M, Morisse S, Sparla F, Perez-Perez ME, Francia F, Danon A, Marchand CH, Fermani S, Trost P, Lemaire SD (2013). Redox regulation of the Calvin-Benson cycle: something old, something new. Front Plant Sci.

[CR16] Muller-Moule P, Conklin PL, Niyogi KK (2002). Ascorbate deficiency can limit violaxanthin de-epoxidase activity in vivo. Plant Physiol.

[CR17] Naranjo B, Mignée C, Krieger-Liszkay A, Hornero-Méndez D, Gallardo-Guerrero L, Cejudo FJ, Lindahl M (2016). The chloroplast NADPH thioredoxin reductase C, NTRC, controls non-photochemical quenching of light energy and photosynthetic electron transport in Arabidopsis. Plant Cell Environ.

[CR18] Nilkens M, Kress E, Lambrev P, Miloslavina Y, Muller M, Holzwarth AR, Jahns P (2010). Identification of a slowly inducible zeaxanthin-dependent component of non-photochemical quenching of chlorophyll fluorescence generated under steady-state conditions in Arabidopsis. Biochim Biophys Acta.

[CR19] Niyogi KK, Grossman AR, Bjorkman O (1998). Arabidopsis mutants define a central role for the xanthophyll cycle in the regulation of photosynthetic energy conversion. Plant Cell.

[CR20] Okegawa Y, Motohashi K (2020). M-type thioredoxins regulate the PGR5/PGRL1-dependent pathway by forming a disulfide-linked complex with PGRL1. Plant Cell.

[CR21] Porra RJ, Thompson WA, Kriedemann PE (1989). Determination of accurate extinction coefficients and simultaneous-equations for assaying chlorophyll-a and chlorophyll-b extracted with 4 different solvents - verification of the concentration of chlorophyll standards by atomic-absorption spectroscopy. Biochim Biophys Acta.

[CR22] Ruban AV (2016). Nonphotochemical chlorophyll fluorescence quenching: mechanism and effectiveness in protecting plants from photodamage. Plant Physiol.

[CR23] Ruban AV, Johnson MP, Duffy CD (2012). The photoprotective molecular switch in the photosystem II antenna. Biochim Biophys Acta.

[CR24] Sekiguchi T, Yoshida K, Wakabayashi KI, Hisabori T (2022). Dissipation of the proton electrochemical gradient in chloroplasts promotes the oxidation of ATP synthase by thioredoxin-like proteins. J Biol Chem.

[CR25] Serrato AJ, Fernandez-Trijueque J, Barajas-Lopez JD, Chueca A, Sahrawy M (2013). Plastid thioredoxins: a "one-for-all" redox-signaling system in plants. Front Plant Sci.

[CR26] Szabo I, Bergantino E, Giacometti GM (2005). Light and oxygenic photosynthesis: energy dissipation as a protection mechanism against photo-oxidation. EMBO Rep.

[CR27] Takahashi S, Murata N (2005). Interruption of the Calvin cycle inhibits the repair of Photosystem II from photodamage. Biochim Biophys Acta.

[CR28] Toivola J, Nikkanen L, Dahlstrom KM, Salminen TA, Lepisto A, Vignols HF, Rintamaki E (2013). Overexpression of chloroplast NADPH-dependent thioredoxin reductase in Arabidopsis enhances leaf growth and elucidates in vivo function of reductase and thioredoxin domains. Front Plant Sci.

[CR29] Yokochi Y, Sugiura K, Takemura K, Yoshida K, Hara S, Wakabayashi KI, Kitao A, Hisabori T (2019). Impact of key residues within chloroplast thioredoxin-f on recognition for reduction and oxidation of target proteins. J Biol Chem.

[CR31] Yokochi Y, Yoshida K, Hahn F, Miyagi A, Wakabayashi KI, Kawai-Yamada M, Weber APM, Hisabori T (2021). Redox regulation of NADP-malate dehydrogenase is vital for land plants under fluctuating light environment. Proc Natl Acad Sci U S A.

[CR30] Yokochi Y, Fukushi Y, Wakabayashi KI, Yoshida K, Hisabori T (2021). Oxidative regulation of chloroplast enzymes by thioredoxin and thioredoxin-like proteins in *Arabidopsis thaliana*. Proc Natl Acad Sci U S A.

[CR32] Yoshida K, Hisabori T (2023). Current insights into the redox regulation network in plant chloroplasts. Plant Cell Physiol.

[CR33] Yoshida K, Matsuoka Y, Hara S, Konno H, Hisabori T (2014). Distinct redox behaviors of chloroplast thiol enzymes and their relationships with photosynthetic electron transport in *Arabidopsis thaliana*. Plant Cell Physiol.

[CR34] Yoshida K, Hara S, Hisabori T (2015). Thioredoxin selectivity for thiol-based redox regulation of target proteins in chloroplasts. J Biol Chem.

[CR35] Yoshida K, Hara A, Sugiura K, Fukaya Y, Hisabori T (2018). Thioredoxin-like2/2-Cys peroxiredoxin redox cascade supports oxidative thiol modulation in chloroplasts. Proc Natl Acad Sci U S A.

[CR36] Yoshida K, Uchikoshi E, Hara S, Hisabori T (2019). Thioredoxin-like2/2-Cys peroxiredoxin redox cascade acts as oxidative activator of glucose-6-phosphate dehydrogenase in chloroplasts. Biochem J.

[CR37] Yoshida K, Yokochi Y, Hisabori T (2019). New light on chloroplast redox regulation: Molecular mechanism of protein thiol oxidation. Front Plant Sci.

